# Maternal and Child Supplementation with Lipid-Based Nutrient Supplements, but Not Child Supplementation Alone, Decreases Self-Reported Household Food Insecurity in Some Settings

**DOI:** 10.3945/jn.117.257386

**Published:** 2017-10-04

**Authors:** Katherine P Adams, Emmanuel Ayifah, Thokozani E Phiri, Malay K Mridha, Seth Adu-Afarwuah, Mary Arimond, Charles D Arnold, Joseph Cummins, Sohrab Hussain, Chiza Kumwenda, Susana L Matias, Ulla Ashorn, Anna Lartey, Kenneth M Maleta, Stephen A Vosti, Kathryn G Dewey

**Affiliations:** 1 Departments of Nutrition and; 2 Agricultural and Resource Economics, University of California, Davis, Davis, CA;; 3 Department of Economics, School of Economic and Business Sciences, University of the Witwatersrand, Johannesburg, South Africa;; 4 School of Public Health and Health Systems, University of Waterloo, Waterloo, Ontario, Canada;; 5 James P Grant School of Public Health, BRAC University, Dhaka, Bangladesh;; 6 Department of Nutrition and Food Science, University of Ghana, Legon, Accra, Ghana;; 7 Department of Economics, University of California, Riverside, Riverside, CA;; 8 Saving Newborn Lives Program, Save the Children International, Dhaka, Bangladesh;; 9 Department of Community Health and; 10 School of Public Health and Family Medicine, University of Malawi College of Medicine, Blantyre, Malawi; and; 11 Center for Child Health Research, University of Tampere Faculty of Medicine and Life Sciences and Tampere University Hospital, Tampere, Finland

**Keywords:** lipid-based nutrient supplements, food insecurity, Bangladesh, Ghana, Malawi

## Abstract

**Background:** It is unknown whether self-reported measures of household food insecurity change in response to food-based nutrient supplementation.

**Objective:** We assessed the impacts of providing lipid-based nutrient supplements (LNSs) to women during pregnancy and postpartum and/or to their children on self-reported household food insecurity in Malawi [DOSE and DYAD trial in Malawi (DYAD-M)], Ghana [DYAD trial in Ghana (DYAD-G)], and Bangladesh [Rang-Din Nutrition Study (RDNS) trial].

**Methods:** Longitudinal household food-insecurity data were collected during 3 individually randomized trials and 1 cluster-randomized trial testing the efficacy or effectiveness of LNSs (generally 118 kcal/d). Seasonally adjusted Household Food Insecurity Access Scale (HFIAS) scores were constructed for 1127 DOSE households, 732 DYAD-M households, 1109 DYAD-G households, and 3671 RDNS households. The impact of providing LNSs to women during pregnancy and the first 6 mo postpartum and/or to their children from 6 to 18–24 mo on seasonally adjusted HFIAS scores was assessed by using negative binomial models (DOSE, DYAD-M, and DYAD-G trials) and mixed-effect negative binomial models (RDNS trial).

**Results:** In the DOSE and DYAD-G trials, seasonally adjusted HFIAS scores were not different between the LNS and non-LNS groups. In the DYAD-M trial, the average household food-insecurity scores were 14% lower (*P* = 0.01) in LNS households than in non-LNS households. In the RDNS trial, compared with non-LNS households, food-insecurity scores were 17% lower (*P* = 0.02) during pregnancy and the first 6 mo postpartum and 15% lower (*P* = 0.02) at 6–24 mo postpartum in LNS households.

**Conclusions:** The daily provision of LNSs to mothers and their children throughout much of the “first 1000 d” may improve household food security in some settings, which could be viewed as an additional benefit that may accrue in households should policy makers choose to invest in LNSs to promote child growth and development. These trials were registered at clinicaltrials.gov as NCT00945698 (DOSE) NCT01239693 (DYAD-M), NCT00970866 (DYAD-G) and NCT01715038 (RDNS).

## Introduction

Food security, in its most basic sense, is the realization of the fundamental human right to sufficient food. Beneath the surface of this basic notion, food security is a complex, multidimensional concept. Set forth at the World Food Summit of 1996, food security is defined as a state in which, “… all people, at all times, have physical and economic access to sufficient, safe and nutritious food that meets their dietary needs and food preferences for an active and healthy life” (1). This definition suggests that to ensure food security food must be available, accessible, and properly utilized consistently over time in a manner that is in line with social and cultural preferences. Food insecurity, then, is the absence of ≥1 of those conditions.

Reliable measures are crucial to monitoring and addressing food insecurity and for targeting and evaluating programs and policies that may affect it (2, 3). Because of the multidimensionality of food security, its measurement is complex, and there is no single gold standard measure (3–5). The Household Food Insecurity Access Scale (HFIAS) is one method developed to measure the household-level “access” dimension of food insecurity or whether households have sufficient resources to obtain the quantity and quality of food needed to support a nutritious diet (6). It is a self-reported, experience-based measure of household-level access to food that combines behavioral and psychological responses to inadequate access to food (2). The HFIAS has been validated in several developing-country settings, including Tanzania (7), Burkina Faso (8), and Ethiopia (9), although it is not recommended for crosscultural comparisons (10).

This article describes the impacts of the provision of lipid-based nutrient supplements (LNSs) given to women during pregnancy and the first 6 mo postpartum and/or to their children on household food insecurity as measured by the HFIAS. The results are based on analyses of longitudinal food-security data that were collected during 4 randomized trials: 2 efficacy trials in Malawi and 1 efficacy trial in Ghana, all conducted by the International Lipid-based Nutrient Supplement (iLiNS) Project research consortium, and 1 effectiveness trial in Bangladesh, the Rang-Din Nutrition Study (RDNS).

These studies, and others testing the efficacy or effectiveness of LNSs, have shown mixed results in terms of child growth and development (11). With regard to the 4 randomized trials included in this article, in the iLiNS DOSE trial in Malawi, child supplementation with LNSs from 6 to 18 mo of age did not affect length gain or prevent stunting (12), nor did it affect child development (13). In the iLiNS DYAD trial in Malawi (DYAD-M), maternal supplementation with LNSs during pregnancy through the first 6 mo postpartum and child supplementation with LNSs from 6 to 18 mo of age did not affect birth outcomes (14), child growth (15), or development (16). In the iLiNS DYAD trial in Ghana (DYAD-G), however, maternal and child supplementation with LNSs increased birth size, particularly among first-time mothers (17) and maternal-plus-child supplementation increased the mean attained length at 18 mo of age (18), although child development at 18 mo of age was not affected (19). Finally, the RDNS effectiveness trial in Bangladesh showed that maternal supplementation with LNSs improved several birth outcomes, including mean birth weight, length, and head circumference, and reduced the prevalence of newborn stunting (20), and maternal-plus-child supplementation improved child growth and development through 24 mo of age (21, 22).

The energy contributions of the LNSs provided through these interventions were relatively small (∼55–241 kcal/d for the DOSE trial and 118 kcal/d for the DYAD-M, DYAD-G, and RDNS trials), and the supplements were intended to fortify, not replace, foods consumed as part of the regular diet. And although the primary hypotheses of the randomized trials were specific to birth outcomes and/or child growth and development, several features of the provision of LNSs raised the possibility that they may have improved the access dimension of household food insecurity. In particular, the food-based supplements were provided regularly and reliably over extended periods of time (daily for between 1 and >2 y), they were of high nutritional quality, and they were provided specifically to nutritionally vulnerable members of the households (pregnant and breastfeeding women and young children). This article reports results of crosscountry analyses to determine whether the provision of LNSs through these interventions influenced household food insecurity as measured by the HFIAS score.

## Methods

### 

#### Study designs.

The study designs for the 4 randomized trials have been described elsewhere in detail (12, 15, 18, 20) and are summarized in **Table 1**. The DOSE trial was designed to test the efficacy of various doses and formulations of LNSs for promoting child growth. Rolling enrollment of children was conducted from November 2009 to May 2011. At ∼6 mo of age, children were randomly assigned to 1 of 5 intervention groups or a delayed-intervention control group. Children in the intervention groups received daily LNSs for 12 mo in one of the following doses and formulations: *1*) 10 g LNS containing milk powder, *2*) 20 g LNS without milk powder, *3*) 20 g LNS containing milk powder, *4*) 40 g LNS without milk powder, or *5*) 40 g LNS containing milk powder. The delayed-intervention control group received no supplementation during the 12-mo intervention period. All children in the trial, regardless of intervention group, received weekly morbidity surveillance and referral by study staff.

**TABLE 1:** Study designs^1^

Trial	DOSE	DYAD-M	DYAD-G	RDNS
Site	Mangochi District, southern Malawi	Mangochi District, southern Malawi	Yilo Krobo and Lower Manya Krobo districts, Eastern Region, Ghana	Badarganj and Chirirbandar subdistricts, northwest Bangladesh
Randomization	Individual child	Individual mother	Individual mother	Cluster, defined by community health workers’ supervision areas
Intervention period	6–18 mo	<20 wk gestation to 18 mo postpartum	<20 wk gestation to 18 mo postpartum	≤20 wk gestation to 24 mo postpartum
Intervention groups	LNS-10gM	LNS: 20 g LNS/d for women during pregnancy and first 6 mo postpartum; 20 g LNS/d for their children from 6 to 18 mo old	LNS: 20 g LNS/d for women during pregnancy and first 6 mo postpartum; 20 g LNS/d for their children from 6 to 18 mo old	Comprehensive LNS: 20 g LNS/d for women during pregnancy and first 6 mo postpartum; 20 g LNS/d for their children from 6 to 24 mo old
	LNS-20g	MMN: Daily multiple micronutrient capsules for women during pregnancy and first 6 mo postpartum; no child supplementation	MMN: Daily MMN capsules for women during pregnancy and first 6 mo postpartum; no child supplementation	Child-only LNS: daily IFA capsules for women during pregnancy and every other day for first 3 mo postpartum; 20 g LNS/d for their children from 6 to 24 mo old
	LNS-20gM	IFA: Daily IFA capsules for women during pregnancy; daily 200 mg Ca placebo for first 6 mo postpartum; no child supplementation	IFA: Daily IFA capsules for women during pregnancy; daily 200 mg Ca placebo for first 6 mo postpartum; no child supplementation	Child-only MNP: daily IFA capsules for women during pregnancy and every other day for first 3 mo postpartum; daily MNP for their children from 6 to 24 mo old
	LNS-40g	—	—	Control: daily IFA capsules for women during pregnancy and every other day for first 3 mo postpartum; no child supplementation
	LNS-40gM			
	Control: delayed intervention control			
Number enrolled	1932	869	1320	4011
Number in food-insecurity analysis	1360	776	1214	3747
Combined groups	LNS: LNS-10gM + LNS-20g + LNS-20gM + LNS-40g + LNS-40gM	LNS: LNS	LNS: LNS	Maternal LNS: comprehensive LNS^2^
	No LNS: control	No LNS: MMN + IFA	No LNS: MMN + IFA	No maternal LNS: child-only LNS + child-only MNP + control^2^
				Child LNS: comprehensive LNS + child-only LNS^3^
				No child LNS: child-only MNP + control^3^

1 DYAD-G, DYAD trial in Ghana; DYAD-M, DYAD trial in Malawi; IFA, iron-folic acid; LNS, lipid-based nutrient supplement; LNS-10gM, 10 g lipid-based nutrient supplement/d with milk; LNS-20g, 20 g lipid-based nutrient supplement/d without milk; LNS-20gM, 20 g lipid-based nutrient supplement/d with milk; LNS-40g, 40 g lipid-based nutrient supplement/d without milk; LNS-40gM, 40 g lipid-based nutrient supplement/d with milk; MMN, multiple micronutrient; MNP, micronutrient powder; RDNS, Rang-Din Nutrition Study.

2 Combination 1 includes periods 1 and 2, which spanned the portion of the intervention from birth of the child through the end of maternal supplementation.

3 Combination 2 includes periods 3–5, which spanned the child supplementation portion of the intervention.

A pair of randomized controlled trials known as the DYAD trials were conducted in Malawi (DYAD-M) and Ghana (DYAD-G) to test the efficacy of LNSs provided to women during pregnancy and the first 6 mo postpartum and to children from 6 to 18 mo of age on birth outcomes and child growth. Rolling enrollment of pregnant women who were at <20 wk gestation occurred between February 2011 and August 2012 in DYAD-M and between December 2009 and December 2011 in DYAD-G. In both trials, women were randomly assigned to 1 of 3 intervention groups. During pregnancy and the first 6 mo postpartum, women in one group received a daily dose of 20 g LNS product designed for maternal consumption, and from 6 to 18 mo of age, the children of women in that group also received a daily dose of 20 g LNS product designed for children. Another group received a daily multiple micronutrient capsule during pregnancy and the first 6 mo postpartum (no child supplementation). A third group received a daily iron-folic acid (IFA) capsule during pregnancy, which is a component of the standard of antenatal care in Ghana and Malawi, and a daily 200-mg Ca placebo for the first 6 mo postpartum (no child supplementation). All mothers and children participating in the DYAD-M and DYAD-G trials also received biweekly (mothers) or weekly (children) morbidity surveillance and referral by study staff.

The RDNS effectiveness trial in Bangladesh was implemented in partnership with LAMB, a nongovernmental organization that provided community-based health services to women and children in the study area. RDNS was a cluster-randomized trial with 4 intervention groups, with clusters defined based on the LAMB community health workers’ 64 work areas. Rolling enrollment of pregnant women who were at ≤20 wk gestation occurred between October 2011 and August 2012. Women randomly assigned to the comprehensive LNS group received 20 g LNS/d through pregnancy and the first 6 mo postpartum, and their children received 20 g LNS/d from 6 to 24 mo of age. Another group of women received daily IFA capsules during pregnancy and the first 3 mo postpartum, and their children received LNSs from 6 to 24 mo. A third group also received IFA capsules during pregnancy and the first 3 mo postpartum, and their children received micronutrient powder from 6 to 24 mo. A final group served as the control and received IFA during pregnancy and the first 3 mo postpartum (no child supplementation). All supplements were delivered by LAMB community health workers.

The combined groups in Table 1 are based on pooling the intervention groups into 2 groups/trial: one group that received LNS and another that did not. In this article, the primary analysis was conducted with the combined groups, and a secondary analysis with the original assigned intervention groups was conducted as a sensitivity analysis.

All trials received ethical approval and were registered at clinicaltrials.gov. The DOSE (NCT00945698) and DYAD-M (NCT01239693) trials were approved by the Research and Ethics Committee of the University of Malawi College of Medicine and by the Ethics Committee of Pirkanmaa Hospital District, Finland. The DYAD-G trial (NCT00970866) was approved by the ethics committees of the University of California, Davis; the Ghana Health Service; and the University of Ghana Noguchi Memorial Institute for Medical Research. The RDNS trial (NCT01715038) was approved by the ethics committees of the University of California, Davis; the International Center for Diarrheal Disease Research, Bangladesh; and LAMB. The nutrient contents of all capsules and LNS products used in the 4 trials are available in **Supplemental Tables 1–3**.

#### Study sites and participants.

Both the DOSE and DYAD-M trials were conducted in the Mangochi District in southern Malawi. DOSE participants were drawn from the semi-urban catchment area of the Mangochi district hospital and the rural catchment area of the Namwera health center. Women were recruited into the DYAD-M trial from the Malindi hospital (semi-urban) and Lungwena health center (rural) in addition to the 2 DOSE recruitment sites. The majority of the population in the Malawi study catchment areas were subsistence farmers and fishers. In Ghana, women were recruited from 4 health facilities operating along a busy commercial corridor running through the Yilo Krobo and Lower Manya Krobo districts in the Eastern Region of the country. The livelihoods of the population in the catchment area were largely supported through petty trade, operating shops and kiosks, and providing skills and services. The RDNS study population was drawn from 11 rural unions of the Badarganj and Chirirbandar subdistricts of the northwest region of Bangladesh where the population was primarily engaged in farming, petty trade, transportation, and construction.

#### Data.

The HFIAS survey instrument, which was developed in generic form by the Food and Nutrition Technical Assistance Project (6), was adapted to each local setting. After enrollment, the adapted questionnaire was administered 2 times to each household in the DOSE trial, 4 times during DYAD-M, 3 times during the DYAD-G trial, and 5 times during the RDNS trial. These self-reported food-insecurity data were collected at multiple time points to document the food-insecurity context for each household at each stage during the intervention, which spanned multiple seasons and, in some cases, multiple years. The adapted survey questions for each trial are available in **Supplemental Methods 1**.

In the DOSE trial, the survey respondent was, in almost all cases, the mother or primary caregiver of the child enrolled in the trial. In the DYAD-M, DYAD-G, and RDNS trials, the survey respondent was the mother enrolled in the trial in almost all cases. For each of 9 food-insecurity access conditions, the survey respondent was asked whether anyone in her household had experienced the condition in the previous 4 wk. If yes, the respondent then indicated how frequently the condition occurred, where “rarely” was 1–2 times in the past 4 wk, “sometimes” was 3–10 times in the past 4 wk, and “often” was >10 times in the past 4 wk. The HFIAS score, a measure of the degree of food insecurity ranging from 0 to 27, was then calculated as the simple sum of the frequency-of-occurrence responses, where “never” was 0 points, “rarely” was 1 point, “sometimes” was 2 points, and “often” was 3 points.

After the HFIAS questions were administered, respondents were then asked about strategies used to cope with food insecurity. The specific coping strategies were developed by using a subset of the generic strategies (23) and locally adapted through focus group discussions conducted at each site. The full text of the coping strategy questions, which were administered at each round of food-security data collection for the DOSE, DYAD-M, and DYAD-G trials and at 2 rounds of food-security data collection for RDNS, are available in **Supplemental Methods 2**.

For the DOSE, DYAD-M, and DYAD-G trials (and to a much lesser extent for the RDNS trial), at each round of food-security data collection, there was substantial variation in the actual timing of data collection visits relative to when the visits were scheduled to occur. To compare food-security observations across households with a similar duration of exposure to the intervention in our analyses, instead of grouping food-security observation by round of data collection, observations were grouped by period, where each period represented a block of time relative to the age of the child enrolled in the trial (**Table 2**).

**TABLE 2:** Food-security data collection periods and sample sizes^1^

Study and child age, mo	*n*
DOSE	
11–15.9	1127
16–18	785
DYAD-M	
0–4.9	732
5–10.9	621
11–15.9	658
≥16	663
DYAD-G	
0–4.9	1109
5–10.9	1048
≥11	983
RDNS	
Baseline	4008
0–4.9	3671
5–6.9	3534
11–12.9	3445
16–18.9	3418
≥23	3438

1 DYAD-G, DYAD trial in Ghana; DYAD-M, DYAD trial in Malawi; RDNS, Rang-Din Nutrition Study.

Women and children were randomly allocated to intervention groups across seasons during the rolling enrollment periods of each trial, but to account for possible imbalances across seasons in subsequent periods of food-security data collection, a seasonally adjusted HFIAS score was constructed. Seasons were identified using cropping calendars and personal communication with local contacts at each site, and seasons were defined as season by year (e.g., the lean season in 1 y was coded separately from the lean season in the following year) to allow for annual variation in seasonal food insecurity. With periods defined as in Table 2 corresponding to the child’s age, the seasonally adjusted HFIAS score for household *i* in season *s* and period *p*, was then defined in Equation *1* as:


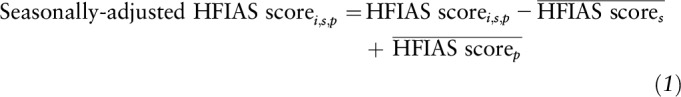


where 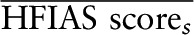 was the average HFIAS score within the control group (IFA group in the case of the DYAD trials) in season *s*, and 
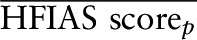
 was the average HFIAS score within the control group (IFA group for DYAD trials) in period *p*. To preserve the integer nature of the score, seasonally adjusted HFIAS scores were rounded to the nearest integer, and negative scores were rounded to zero.

#### Statistical methods.

The analyses were conducted by intent-to-treat and were performed separately for each trial. RDNS data from periods 1 and 2 were also analyzed separately from periods 3–5 because the combined intervention groups (described in Table 1) differed between the 2 sets of periods. Households with missed food-security visits were included in the analysis for all time points where data were available. In cases in which a food-security visit occurred far off schedule, resulting in 2 observations for the same household in one period, the visit closest to the scheduled date during that period was retained, and the other observation was dropped from the analysis. Analyses were conducted by using Stata 14 (StataCorp).

The seasonally -adjusted HFIAS scores are essentially count data, and for all trials the distribution of scores was positively skewed. The effects of the DOSE, DYAD-M, and DYAD-G interventions on household food insecurity were therefore estimated by using negative binomial models with a household-level robust variance estimation to account for repeated measures. The RDNS models were estimated by using mixed-effect negative binomial models with random effects at 3 levels to account for the cluster design and the repeated measures: households nested within community health worker work areas, work areas nested within regional unions, and unions. All models included fixed effects for the period of food-security data collection. For the DOSE, DYAD-M, and DYAD-G trials, the scheduled baseline round of food-security data collection was done after random assignment for many, but not all, households, so the baseline round was omitted from those analyses. The baseline round was collected before random assignment for all RDNS households and was therefore included in all of the RDNS analyses as a covariate control.

For all analyses, when the null hypothesis of no difference between intervention groups was rejected (*P* < 0.05), pairwise comparison incidence rate ratios, which are ratios of predicted seasonally adjusted HFIAS scores in the LNS intervention groups to the predicted scores in the non-LNS groups, were estimated and are referred to as predicted score ratios (PSRs). For analyses with >2 intervention groups, *P* values for post hoc pairwise comparisons of PSRs were adjusted for multiple comparisons by using Sidak’s method (24). Interaction terms between intervention group and period of data collection were used to assess differences in the effect of intervention group by period and were further examined by estimating the group marginal means for each period.

Models that included an additional set of prespecified baseline covariates were estimated to determine whether adjusting for the additional covariates improved the precision of the estimated effects (25). Baseline covariates were included in the fully adjusted models if they were associated with the seasonally adjusted HFIAS score at the 10% level of significance in bivariate analyses. For all trials, season of food-security data collection, maternal age, and level of education and household electrification were included in fully adjusted models. For DOSE, maternal marital status and household distance to a main market were also included in fully adjusted models. DYAD-M and DYAD-G adjustment covariates also included maternal parity and household distance to a main market, and DYAD-M additionally included maternal marital status. In addition to the baseline seasonally adjusted HFIAS score, which was included in all RDNS models, RDNS fully adjusted models also included maternal parity and maternal BMI (in kg/m^2^). Effect modification by each baseline covariate was assessed by including a group-by-covariate interaction term.

When seasonally adjusted HFIAS scores were significantly different between intervention groups, secondary analyses of individual HFIAS questions and food-insecurity coping strategies were performed to understand the drivers of the effect. Responses to individual HFIAS questions as well as responses to questions about specific food-insecurity coping strategies were coded as dichotomous variables and analyzed by using logistic models with household-level robust variance or mixed-effect logistic models with random effects of union, cluster, and household.

## Results

### 

#### Baseline characteristics and balance.

For all trials, maternal and household characteristics (**Table 3**) were similar between combined intervention groups in the food-security samples with the exception of maternal education and maternal parity in the RDNS sample. Maternal education was slightly higher (6.4 ± 3.2 y compared with 6.1 ± 3.3 y, *P* = 0.02) in the maternal LNS group than in the maternal group with no LNS, and it was higher (6.4 ± 3.3 y compared with 6.1 ± 3.2 y, *P* = 0.01) in the child LNS group than in the child group with no LNSs. Mothers in the RDNS sample whose children received LNSs were also more likely to be nulliparous at baseline than were mothers whose children did not receive LNSs (42% compared with 38%, *P* = 0.03).

**TABLE 3:** Baseline characteristics among households of the women who participated in randomized trials in Malawi (DOSE and DYAD-M), Ghana (DYAD-G), and Bangladesh (RDNS)^1^

	DOSE (*n* = 1099)	DYAD-M (*n* = 755)	DYAD-G (*n* = 1181)	RDNS (*n* = 3737)
Maternal age, y	25.8 ± 6.4	24.8 ± 6.1	26.9 ± 5.5	21.9 ± 5.0
Maternal education, y	4.5 ± 3.5	3.9 ± 3.5	7.4 ± 3.7	6.2 ± 3.2
Maternal BMI, kg/m^2^	21.8 ± 2.9	22.0 ± 2.8	24.8 ± 4.6	20.0 ± 2.6
Mother married, %	87.7	89.4	NA	99.9
Mother nulliparous at baseline, %	NA	21.7	33.7	40.2
Household has electricity, %	7.3	7.7	85.0	36.2
Household distance to market, km	3.4 ± 2.8	2.0 ± 2.8	1.9 ± 1.8	N/A
Baseline seasonally adjusted HFIAS score	NA	NA	NA	1 (0, 5)

1 Values are means ± SDs or median (25th, 75th percentiles) unless otherwise indicated. DYAD-G, DYAD trial in Ghana; DYAD-M, DYAD trial in Malawi; HFIAS, Household Food Insecurity Access Scale; NA, variable was not available; RDNS, Rang-Din Nutrition Study.

#### Effects on household food insecurity.

**Table 4** shows seasonally adjusted HFIAS scores by combined intervention group as estimated marginal means or average predicted group means adjusted for other effects in the models. Over the course of all food-security data collection periods, seasonally adjusted HFIAS scores did not differ significantly between the combined intervention groups in the DOSE or DYAD-G trials. In the DYAD-M trial, the PSR was 0.86 and was statistically significant (*P* = 0.01), indicating that household food-insecurity scores were 14% lower in households in which the mother and her child received LNSs than in households that did not receive LNSs. In the RDNS trial during pregnancy and the first 6 mo postpartum (periods 1 and 2), food-insecurity scores were 17% lower (PSR: 0.83, *P* = 0.02) among households in which the mother received LNSs than in those who received IFA through pregnancy and the first 3 mo postpartum. In periods 3–5 of the RDNS trial, food-insecurity scores were 15% lower (PSR: 0.85, *P* = 0.02) in households in which the child received LNSs from 6 to 24 mo of age than in households in which the child did not receive supplementation.

**TABLE 4:** Seasonally adjusted HFIAS scores by combined intervention groups among households of the women and/or children who participated in randomized trials in Malawi (DOSE and DYAD-M), Ghana (DYAD-G), and Bangladesh (RDNS)^1^

	HFIAS score (95% CI)	*P*
DOSE (*n* = 1912)		0.43
LNS	4.35 (4.10, 4.61)	
No LNS	4.12 (3.61, 4.63)	
DYAD-M (*n* = 2674)^2^		0.01
LNS	3.81 (3.47, 4.15)	
No LNS	4.45 (4.16, 4.73)	
DYAD-G (*n* = 3140)		0.86
LNS	1.78 (1.49, 2.08)	
No LNS	1.82 (1.59, 2.05)	
RDNS, periods 1 and 2 (*n* = 7204)^3^		0.02
Maternal LNS	1.60 (1.31, 1.89)	
No maternal LNS	1.94 (1.66, 2.21)	
RDNS, periods 3–5 (*n* = 10,301)^4^		0.02
Child LNS	1.74 (1.38, 2.11)	
No child LNS	2.06 (1.64, 2.49)	

1 Combined intervention groups are defined in Table 1. For DOSE, DYAD-M, and DYAD-G, group values are average marginal mean seasonally adjusted HFIAS scores over all periods of food-security data collection estimated with negative binomial models with household-level robust variance and fixed effects of period of food-security data collection. For RDNS, group values are average marginal mean seasonally adjusted HFIAS scores over indicated periods of food-security data collection estimated with mixed-effect negative binomial models with random effects of household, work area, and union, fixed effects of period of food-security data collection, and adjusted for baseline seasonally adjusted HFIAS score. DYAD-G, DYAD trial in Ghana; DYAD-M, DYAD trial in Malawi; HFIAS, Household Food Insecurity Access Scale; LNS, lipid-based nutrient supplement; PSR, predicted score ratio; RDNS, Rang-Din Nutrition Study.

2 PSR: 0.86, *P* = 0.01.

3 PSR: 0.83, *P* = 0.02.

4 PSR: 0.85, *P* = 0.02.

As a sensitivity analysis, we estimated marginal mean seasonally adjusted HFIAS scores by assigned intervention groups (**Table 5**). There were no differences between intervention groups in the DOSE or DYAD-G trials, but there were significant differences in food-insecurity scores between intervention groups in the DYAD-M trial (*P* = 0.02) and periods 3–5 of the RNDS trial (*P* = 0.04). Pairwise tests showed that in the DYAD-M trial, household food-insecurity scores were 15% lower in the LNS group than in the IFA group (PSR: 0.85, *P* = 0.04), and there was a trend toward a difference between the LNS group and the multiple-micronutrient group (PSR: 0.86, *P* = 0.05). In the RDNS trial, pairwise tests showed that household food-insecurity scores were 25% lower (PSR: 0.75, *P* = 0.03) in the comprehensive LNS group than in the control group. Household food insecurity was not significantly different between any other pairs of intervention groups.

**TABLE 5:** Seasonally-adjusted HFIAS scores by assigned intervention groups among households of the women and/or children who participated in randomized trials in Malawi (DOSE and DYAD-M), Ghana (DYAD-G) and Bangladesh (RDNS)^1^

	HFIAS score (95% CI)	*P*^2^
DOSE (*n* = 1912)		0.47
LNS, 40 g	4.54 (4.14, 4.94)	
LNS, 20 g	4.30 (3.87, 4.72)	
LNS, 10 g	4.07 (3.52, 4.62)	
Control	4.12 (3.61, 4.63)	
DYAD-M (*n* = 2674)^3^		0.02
LNS	3.81 (3.47, 4.15)	
MMN	4.42 (4.04, 4.79)	
IFA	4.48 (4.06, 4.90)	
DYAD-G (*n* = 3140)		0.75
LNS	1.78 (1.49, 2.08)	
MMN	1.73 (1.43, 2.03)	
IFA	1.09 (1.56, 2.24)	
RDNS, periods 3–5 (*n* = 10301)^4^		0.04
Comprehensive LNS	1.64 (1.26, 2.01)	
Child-only LNS	1.87 (1.42, 2.31)	
Child-only MNP	1.96 (1.52, 2.41)	
Control	2.17 (1.67, 2.68)	

1 Total sample size over all periods of data collection indicated by “*n*.” Intervention groups defined in Table 1. DOSE intervention groups combine “with” and “without” milk groups at each dosage of LNS. For DOSE, DYAD-M, and DYAD-G, group values are average marginal mean seasonally-adjusted HFIAS scores (95% CIs) over all periods of food-security data collection estimated with negative binomial models with household level robust variance and fixed effects of period of food-security data collection. For RDNS group values are average marginal mean seasonally-adjusted HFIAS scores (95% CIs) over indicated periods of food-security data collection estimated with mixed effect negative binomial models with random effects of household, work area, and union, fixed effects of period of food-security data collection, and adjusted for baseline seasonally-adjusted HFIAS score. HFIAS, Household Food Insecurity Access Scale; IFA, iron-folic acid; LNS, lipid-based nutrient supplement; MMN, multiple micronutrient; MNP, micronutrient powder; PSR, predicted score ratio; RDNS, Rang-Din Nutrition Study.

2 *P* values for Wald tests of joint significance of intervention groups.

3 LNS compared with IFA: PSR = 0.85, *P* = 0.04; MMN compared with IFA: PSR = 0.98, *P* = 0.99; LNS compared with MMN: PSR = 0.86, *P* = 0.05.

4 Comprehensive LNS compared with Control: PSR = 0.75, *P* = 0.03; Child-only LNS compared with Control: PSR = 0.86, *P* = 0.58; Child-only MNP compared with Control: PSR = 0.90, *P* = 0.89; Comprehensive LNS compared with Child-only LNS: PSR = 0.88, *P* = 0.72; Comprehensive LNS compared with Child-only MNP: PSR = 0.83, *P* = 0.34; Child-only LNS compared with Child-only MNP: PSR = 0.95, *P* = 0.99.

**FIGURE 1:**
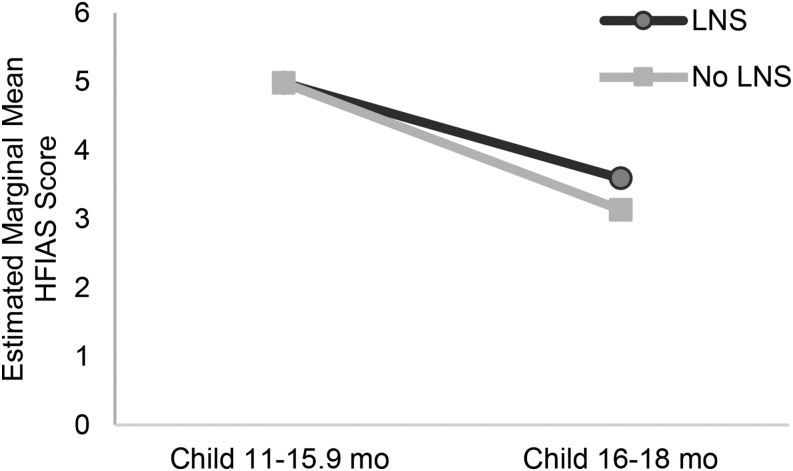
Estimated marginal mean seasonally adjusted HFIAS scores by combined intervention group and period of data collection among households of the children who participated in the DOSE randomized trial in Malawi (*n* = 1912). Estimates are from negative binomial models with household-level robust variance and group-by-period interactions. There was no difference in food insecurity between the group with LNSs and the group without LNSs in either period. HFIAS, Household Food Insecurity Access Scale; LNS, lipid-based nutrient supplement.

**FIGURE 2:**
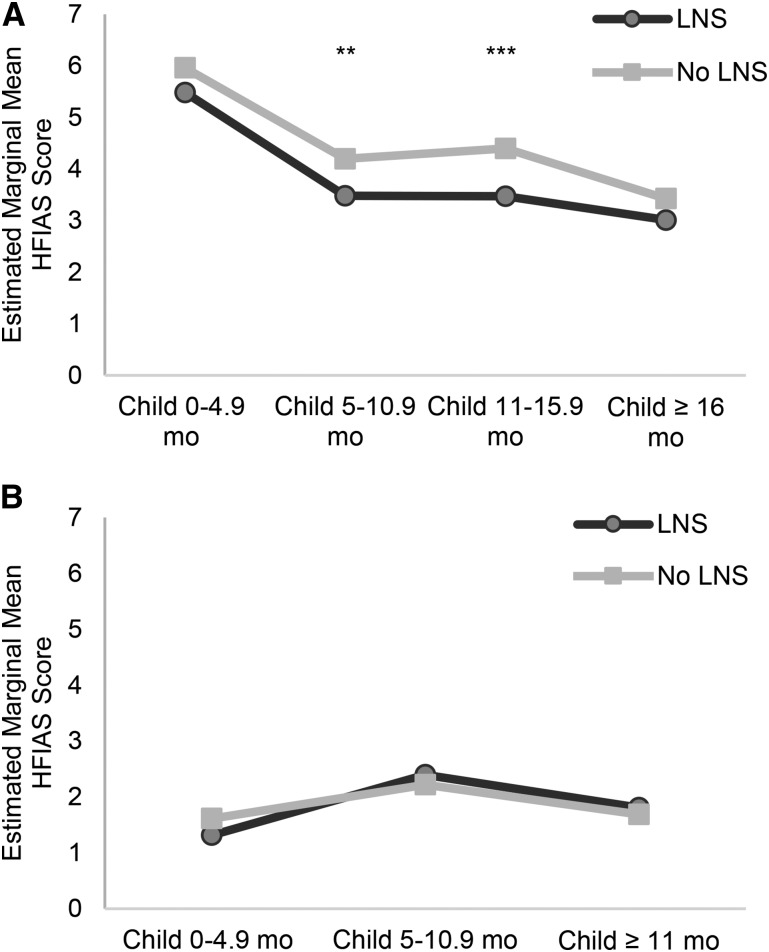
Estimated marginal mean seasonally adjusted HFIAS scores by combined intervention group and period of data collection among households of the women and their children who participated in the DYAD randomized trial in Malawi (*n* = 2674) (A) and the DYAD randomized trial in Ghana (*n* = 3140) (B). All estimates are from negative binomial models with household-level robust variance and group-by-period interactions. **,***PSR between the group with LNSs and the group with no LNSs at the indicated age: **P < 0.05, ***P < 0.01. HFIAS, Household Food Insecurity Access Scale; LNS, lipid-based nutrient supplement; PSR, predicted score ratio.

**FIGURE 3:**
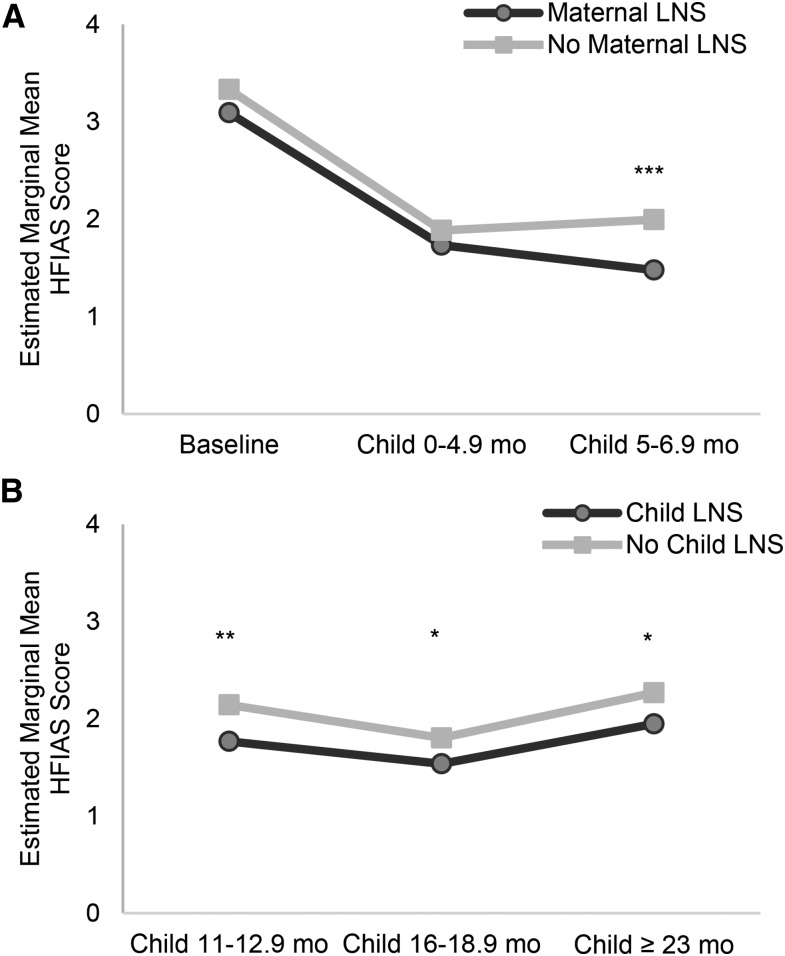
Baseline and periods 1 and 2 (*n* = 7204) (A) and periods 3–5 (*n* = 10,301) (B) estimated marginal mean seasonally adjusted HFIAS scores by combined intervention group and period of data collection among households of the women and their children who participated in the RDNS randomized trial in Bangladesh. Baseline estimates are from mixed-effect negative binomial models with random effects of work area and union. Postbaseline estimates are from mixed-effect negative binomial models adjusted for baseline seasonally adjusted HFIAS score and random effects of union, cluster, and household and group-by-period interactions. *,**,***PSR between the group with LNSs and the group with no LNSs at the indicated age: *P < 0.1, **P < 0.05, ***P < 0.01. HFIAS, Household Food Insecurity Access Scale; LNS, lipid-based nutrient supplement; PSR, predicted score ratio; RDNS, Rang-Din Nutrition Study.

**Figures 1–3** (and **Supplemental Tables 4–7**) show the estimated marginal mean seasonally adjusted HFIAS scores by combined groups for each period of data collection. There were no significant differences in any period between the LNS group and the groups without LNSs in either the DOSE (Figure 1) or DYAD-G (Figure 2B) trials. In the DYAD-M trial (Figure 2A), household food-insecurity scores were significantly lower among the LNS households compared with the no LNS households when the children in the trial were 5–10.9 mo old (PSR: 0.49, *P* = 0.04) and when the children were 11–15.9 mo old (PSR: 0.39, *P* = 0.002). In the RDNS trial there was no significant difference by group in seasonally adjusted HFIAS scores at baseline or in period 1 when the children were 0–4.9 mo old (Figure 3A), but household food-insecurity scores were significantly lower (PSR: 0.60, *P* = 0.002) in the LNS group when the children were 5–6.9 mo of age. During periods 3–5, when the children in the LNS groups received the supplement directly, food-insecurity scores were significantly lower in the LNS group when the children were 11–12.9 mo old (PSR: 0.96, *P* = 0.03) and marginally significant when they were 16–18.9 mo old (*P* = 0.07) and ≥23 mo old (*P* = 0.08).

Adjusting for additional baseline covariates (**Supplemental Tables 8–13**) did not generally improve the precision of the estimated effects and resulted in qualitatively similar results, although the estimated differences between RDNS intervention groups were attenuated. Interaction tests to examine heterogeneity in the effect of receiving LNS on household food insecurity revealed no modification by any of the prespecified potential effect modifiers in any of the trials.

#### Drivers of the effect.

The HFIAS questions captured 3 domains of food insecurity. The first, anxiety and uncertainty about the household food supply, was captured by asking whether the respondent worried that the household would not have enough food. The second was insufficient dietary quality, which was captured by asking about the lack of resources leading to an inability to eat preferred foods, the need to eat a limited variety of foods, and eating unwanted foods. Finally, the third domain, insufficient food intake and its physical consequences, was captured by asking about eating smaller meals than respondents felt were needed, eating fewer meals in a day, having no food in the household, going to sleep at night hungry, and going a full day and night without eating. To understand which aspects of food insecurity in particular were influenced by the receipt of LNSs by households in the DYAD-M and RDNS trials, we estimated the effect of combined intervention group over the course of all food-security data collection periods on the probability of experiencing each of the 9 food-insecurity access conditions at least once in the 4-wk recall period.

**FIGURE 4:**
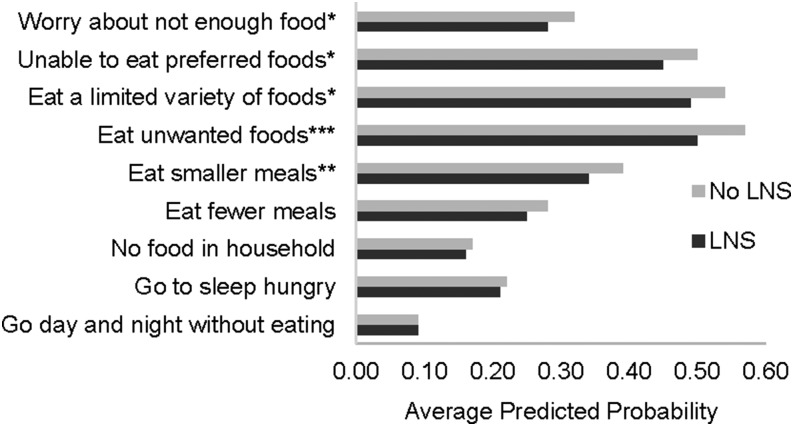
Average predicted probability over all periods of food-security data collection of experiencing the food-insecurity access condition ≥1 time in the 4-wk recall period among households of the women and their children who participated in the DYAD randomized trial in Malawi (*n* = 2674). Predicted probabilities were estimated by using logistic models with household-level robust variance. *,**,***Difference in the predicted probability between the group with LNSs and the group with no LNSs: *P < 0.1, **P < 0.05, ***P < 0.01. LNS, lipid-based nutrient supplement.

In the DYAD-M trial (**Figure 4**), the probability of worrying about not enough food was significantly lower in the group that received LNSs than in the group that did not. In terms of insufficient quality, households that received LNSs had lower probabilities of being unable to eat preferred foods, eating a limited variety of foods, and having to eat unwanted foods than did households that did not receive LNSs. And finally of the questions pertaining to insufficient food intake, households that received LNSs had a lower probability of eating smaller meals than did households that did not receive LNSs.

**FIGURE 5:**
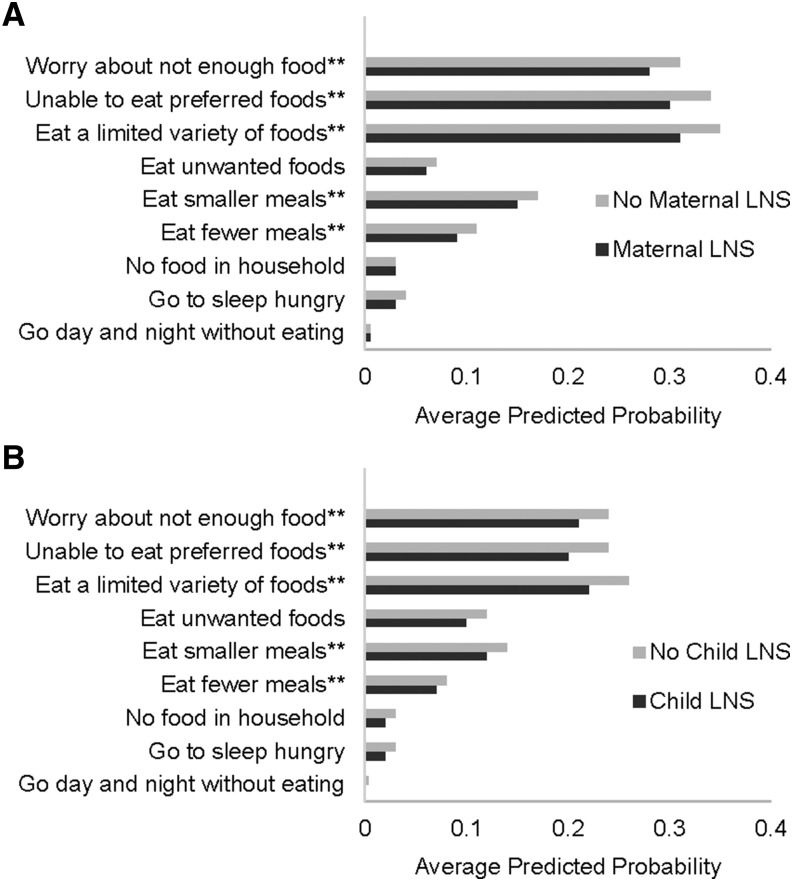
Average predicted probability across periods 1 and 2 (*n* = 7210) (A) and periods 3–5 (*n* = 10,310) (B) of experiencing the food-insecurity access condition ≥1 time in the 4-wk recall period among households of the women and their children who participated in the Rang-Din Nutrition Study randomized trial in Bangladesh. Predicted probabilities were estimated by using logistic mixed models with random effects of household, work area, and union. **Difference in predicted probability between the maternal (child) group with LNSs and the maternal (child) group with no LNSs, **P < 0.05. LNS, lipid-based nutrient supplement.

In the RDNS trial during periods 1 and 2 and periods 3–5 (**Figure 5**), households that received LNSs for maternal (child) consumption had lower probabilities of worrying about not enough food, being unable to eat preferred foods, eating a limited variety of foods, and eating fewer meals. During periods 1 and 2, households that received LNSs for maternal consumption also had a lower probability of eating fewer meals, and during periods 3–5 households that received LNSs for child consumption were also less likely to eat unwanted foods.

**FIGURE 6:**
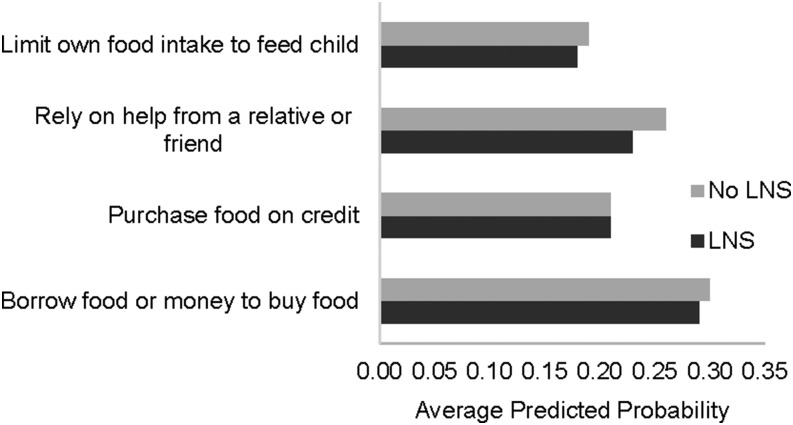
Average predicted probability of relying on a food-insecurity coping strategy ≥1 time in the 4-wk recall period among households of the women and their children who participated in the DYAD randomized trial in Malawi (*n* = 2674). Predicted probabilities were estimated by using logistic models with household-level robust variance. There were no differences in predicted probabilities between groups with and without LNSs. LNS, lipid-based nutrient supplement.

**FIGURE 7:**
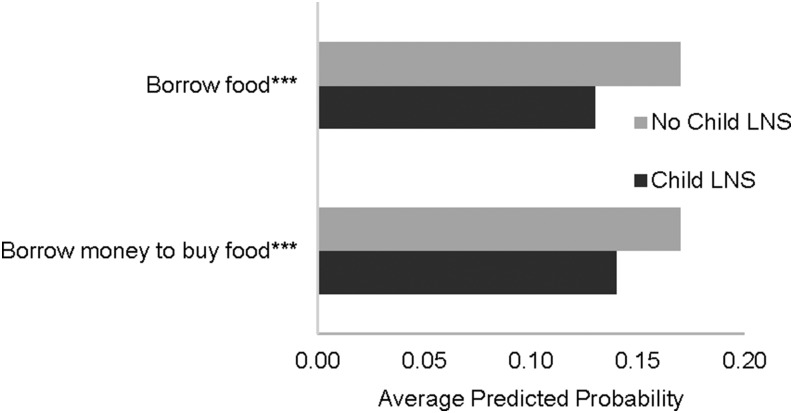
Average predicted probability during periods 3–5 of relying on a food-insecurity coping strategy ≥1 time in the 4-wk recall period among households of women and their children who participated in the Rang-Din Nutrition Study randomized trial of LNS in Bangladesh (*n* = 6889). Predicted probabilities were estimated by using logistic mixed models with random effects of household, work area, and union. ***Difference in predicted probability between the child group with LNSs and the child group with no LNSs, *P* < 0.01. LNS, lipid-based nutrient supplement.

**Figures 6** and **7** show, by combined intervention groups, the average predicted probabilities that households reported relying on each coping strategy at least once in the 4-wk recall period. For DYAD-M, there were no significant group differences in any of these over the course of all food-security data collection periods. In the RDNS trial, only the questions about borrowing money and borrowing food had sufficient variation to warrant statistical analysis. As shown in Figure 7, on average across the 2 periods, households that received LNSs for the child were less likely to borrow food (*P* = 0.001) and less likely to borrow money to buy food (*P* = 0.01) than were households that did not receive LNSs for the child.

## Discussion

The results of these analyses suggest that the provision of LNSs influenced household food insecurity in some contexts but not others. To our knowledge, this is the first study to evaluate the effects of supplementation with a food-based product such as LNSs on an experience-based measure of food insecurity. In the DOSE efficacy trial in Malawi and the DYAD-G efficacy trial, there were no significant differences in food insecurity between households that received LNS and households that did not. However, in the DYAD-M efficacy trial and the RDNS effectiveness trial in Bangladesh, household food insecurity as measured by a seasonally adjusted HFIAS score was lower among households that received LNSs for mothers during pregnancy and the first 6 mo postpartum and for their children from 6 to 18 or 24 mo of age than among households that did not receive LNSs. In both the DYAD-M and RDNS trials, the difference in food insecurity by group appeared to be driven primarily by differences in anxiety and uncertainty about the household food supply and in insufficient food quality.

Bearing in mind that some coping strategies may not be available to the most food-insecure and resource-poor households (such households may lack access to credit, for example, leaving them unable to borrow money to buy food) (6), in the DYAD-M trial the difference in food insecurity did not translate into differences in how households coped with food insecurity. In the RDNS trial, however, households that received LNSs for the child had a lower probability of reporting borrowing food or money to buy food as a means to cope with food shortages in their households.

The variability of our findings across the 4 trials merits discussion. The null effect in the DYAD-G trial is not particularly surprising and is likely explained by the low prevalence of food insecurity among the Ghanaian sample. Because the households that participated in the DOSE and DYAD-M trials were drawn from a very similar population in Malawi, the null effect in the DOSE trial is less easily explained in light of the positive effect in the DYAD-M sample. A key difference between the 2 trials in Malawi was that DYAD-M was comprehensive from pregnancy through 18 mo postpartum, whereas DOSE provided LNSs only to children from 6 to 18 mo of age. Perhaps the continuity of a reliable source of food-based nutrients throughout a large proportion of the first 1000 d provided a nutritional safety-net of sorts that made mothers feel more secure in their ability to meet nutrient needs during this critical period, resulting in less worry about having inadequate food and less need to rely on lower-quality or less-preferred foods. It is also noteworthy that analyses of the impact of LNSs on infant feeding practices showed a similar pattern of effects; the provision of LNSs improved complementary feeding practices in the DYAD-M trial (at 18 mo of age, children who received LNSs were more likely to meet the WHO criterion for frequency of complementary feeding and less likely to have a low frequency of consumption of animal‐source foods in the previous week), but feeding practices were not affected in the DOSE (or DYAD-G) trials (26).

Because the HFIAS score is a self-reported measure of household food insecurity, it is possible that respondents either over- or understated their food-insecurity experiences or that the provision of LNSs changed the nature and extent of that reporting bias. We acknowledge the potential for respondent bias in our measurement of household food insecurity, but this concern is at least partly mitigated by the fact that respondents were asked about food insecurity several times over the span of ≥1 y, making it less likely that respondents may have expected that their answers could affect services provided to the household. A similar concern is that because the respondent to the food-insecurity questionnaire was, in almost all cases, the mother of the child who was receiving LNSs or the mother enrolled in the trial, it is possible (perhaps even likely) that the effects on food insecurity reported here reflect respondents’ personal experiences with food insecurity and may not necessarily reflect effects at the household level. We acknowledge this limitation but also note that improvements in food security among vulnerable household members are encouraging regardless of diffusion throughout the household.

Another limitation is that the HFIAS is not crossculturally valid primarily because of variation in how some concepts in the HFIAS questions (e.g., “worry”) are interpreted in different cultures as well as the crosscultural variation in the degree of food insecurity in which the concepts typically become relevant (10). As such, we cannot compare the HFIAS scores across our samples, nor can we compare the magnitude of the effect of receiving LNSs on the HFIAS score between Malawi and Bangladesh.

A final consideration concerns the external validity of our results. Our findings were generated in the context of efficacy and effectiveness trials where the costs households faced in accessing LNSs were, by design, very low. In the DOSE, DYAD-M, and DYAD-G trials, supplements were regularly delivered to households by study staff, whereas in the RDNS trial, supplements were regularly delivered to households by LAMB community health workers and village health volunteers. It is unlikely that the observed effects on food insecurity would also arise if households faced monetary costs to access LNSs.

We conclude that the daily provision of LNSs throughout much of the first 1000 d improved the access dimension of household food security in Malawi and in Bangladesh. The absolute size of the effect of receiving LNSs on food insecurity was small in both sites, particularly in Bangladesh where levels of food insecurity were relatively low. However, in both trials the LNSs provided only 118 kcal/d, so any detectible effect that persisted over multiple periods during the trials may be noteworthy. Experience-based measures of food insecurity have been shown to be associated with diverse negative outcomes in developing-country settings, such as anxiety and depressive symptoms (27), including specifically among mothers (28) and pregnant women (29), child undernutrition (30, 31), poor antenatal and postnatal maternal dietary diversity (32), and lower subjective wellbeing (33). Given the evidence linking food insecurity to mental health, nutritional status, and behavior, even small improvements in household food security might be beneficial during pregnancy and early childhood.

The role LNSs will play in efforts to prevent early childhood undernutrition is ultimately a policy decision. The results presented here provide new information for decision makers to consider when setting all of the potential benefits of LNSs alongside all of the associated costs. In circumstances where household costs associated with accessing LNSs would be very low (via targeted subsidies, for example), these results suggest that the provision of LNSs has the potential to bring about a small reduction in the prevalence of experience-based food-insecurity conditions, such as worry about insufficient food and inadequate diet quality. Given the magnitude of the effects and the costs associated with providing LNSs, investing in LNSs solely as a means to improve food security would presumably not be cost-effective. Rather, where policy makers chose to invest in LNSs to address child growth and development, improved food security could be viewed as an additional benefit that may accrue in some settings.

## References

[1] FAO. Rome Declaration on World Food Security and World Food Summit Plan of Action [Internet].c1996 [cited 2017 Apr 17]. Available from: http://www.fao.org/docrep/003/w3613e/w3613e00.htm.

[2] Maxwell D, Coates J, Vaitla B How do different indicators of household food security compare? Empirical evidence from Tigray [Internet]. c2013 [cited 2017 Apr 17]. Available from: http://fic.tufts.edu/assets/Different-Indicators-of-HFS.pdf.

[3] Webb P, Coates J, Frongillo EA, Rogers BL, Swindale A, Bilinsky P Measuring household food insecurity: why it’s so important and yet so difficult to do. J Nutr 2006;136:1404S–8S. 16614437 10.1093/jn/136.5.1404S

[4] Barrett CB Measuring food insecurity. Science 2010;327:825–8. 20150491 10.1126/science.1182768

[5] Jones AD, Ngure FM, Pelto G, Young SL What are we assessing when we measure food security? A compendium and review of current metrics. Adv Nutr 2013;4:481–505. 24038241 10.3945/an.113.004119 PMC3771133

[6] Coates J, Swindale A, Bilinsky P Household Food Insecurity Access Scale (HFIAS) for measurement of food access: indicator guide [Internet]. c2007 [cited 2017 Apr 17]. Available from: https://www.fantaproject.org/sites/default/files/resources/HFIAS_ENG_v3_Aug07.pdf.

[7] Knueppel D, Demment M, Kaiser L Validation of the household food insecurity access scale in rural Tanzania. Public Health Nutr 2010;13:360–7. 19706211 10.1017/S1368980009991121

[8] Becquey E, Martin-Prevel Y, Traissac P, Dembélé B, Bambara A, Delpeuch F The household food insecurity access scale and an index-member dietary diversity score contribute valid and complementary information on household food insecurity in an urban West-African setting. J Nutr 2010;140:2233–40. 20962154 10.3945/jn.110.125716

[9] Gebreyesus SH, Lunde T, Mariam DH, Woldehanna T, Lindtjørn B Is the adapted Household Food Insecurity Access Scale (HFIAS) developed internationally to measure food insecurity valid in urban and rural households of Ethiopia? BMC Nutrition 2015;1:1–10.

[10] Deitchler M, Ballard T, Swindale A, Coates J Validation of a measure of household hunger for cross-cultural use [Internet]. c2010 [cited 2017 Apr 17]. Available from: https://www.fantaproject.org/sites/default/files/resources/HHS_Validation_Report_May2010_0.pdf.

[11] Food and Nutrition Technical Assistance III Project (FANTA). Meeting report: evidence and programmatic considerations for the use of small-quantity lipid-based nutrient supplements for the prevention of malnutrition [Internet]. 2016 [cited 2017 Apr 17]. Available from: https://www.fantaproject.org/sites/default/files/resources/SQ-LNS_Meeting_Report_FINAL_Dec2016.pdf.

[12] Maleta KM, Phuka J, Alho L, Cheung YB, Dewey KG, Ashorn U, Phiri N, Phiri TE, Vosti SA, Zeilani M, Provision of 10–40 g/d lipid-based nutrient supplements from 6 to 18 months of age does not prevent linear growth faltering in Malawi. J Nutr 2015;145:1909–15. 26063066 10.3945/jn.114.208181

[13] Prado EL, Phuka J, Maleta K, Ashorn P, Ashorn U, Vosti SA, Dewey KG Provision of lipid-based nutrient supplements from age 6 to 18 months does not affect infant development scores in a randomized trial in Malawi. Matern Child Health J 2016;20:2199–208. 27395385 10.1007/s10995-016-2061-6

[14] Ashorn P, Alho L, Ashorn U, Cheung YB, Dewey KG, Harjunmaa U, Lartey A, Nkhoma M, Phiri N, Phuka J, The impact of lipid-based nutrient supplement provision to pregnant women on newborn size in rural Malawi: a randomized controlled trial. Am J Clin Nutr 2015;101:387–97. 25646337 10.3945/ajcn.114.088617

[15] Ashorn P, Alho L, Ashorn U, Cheung YB, Dewey KG, Gondwe A, Harjunmaa U, Lartey A, Phiri N, Phiri TE, Supplementation of maternal diets during pregnancy and for 6 months postpartum and infant diets thereafter with small-quantity lipid-based nutrient supplements does not promote child growth by 18 months of age in rural Malawi: a randomized controlled trial. J Nutr 2015;145:1345–53. 25926413 10.3945/jn.114.207225

[16] Prado EL, Maleta K, Ashorn P, Ashorn U, Vosti SA, Sadalaki J, Dewey KG Effects of maternal and child lipid-based nutrient supplements on infant development: a randomized trial in Malawi. Am J Clin Nutr 2016;103:784–93. 26843155 10.3945/ajcn.115.114579

[17] Adu-Afarwuah S, Lartey A, Okronipa H, Ashorn P, Zeilani M, Peerson JM, Arimond M, Vosti S, Dewey KG Lipid-based nutrient supplement increases the birth size of infants of primiparous women in Ghana. Am J Clin Nutr 2015;101:835–46. 25833980 10.3945/ajcn.114.091546

[18] Adu-Afarwuah S, Lartey A, Okronipa H, Ashorn P, Peerson JM, Arimond M, Ashorn U, Zeilani M, Vosti S, Dewey KG Small-quantity, lipid-based nutrient supplements provided to women during pregnancy and 6 mo postpartum and to their infants from 6 mo of age increase the mean attained length of 18-mo-old children in semi-urban Ghana: a randomized controlled trial. Am J Clin Nutr 2016;104:797–808. 27534634 10.3945/ajcn.116.134692 PMC4997301

[19] Prado EL, Adu-Afarwuah S, Lartey A, Ocansey M, Ashorn P, Vosti SA, Dewey KG Effects of pre- and post-natal lipid-based nutrient supplements on infant development in a randomized trial in Ghana. Early Hum Dev 2016;99:43–51. 27391572 10.1016/j.earlhumdev.2016.05.011

[20] Mridha MK, Matias SL, Chaparro CM, Paul RR, Hussain S, Vosti SA, Harding KL, Cummins JR, Day LT, Saha SL, Lipid-based nutrient supplements for pregnant women reduce newborn stunting in a cluster-randomized controlled effectiveness trial in Bangladesh. Am J Clin Nutr 2016;103:236–49. 26607935 10.3945/ajcn.115.111336 PMC6443293

[21] Matias SL, Mridha MK, Tofail F, Arnold CD, Khan MSA, Siddiqui Z, Ullah MB, Dewey KG Home fortification during the first 1000 d improves child development in Bangladesh: a cluster-randomized effectiveness trial. Am J Clin Nutr 2017;105:958–69. 28275128 10.3945/ajcn.116.150318

[22] Dewey KG, Mridha MK, Matias SL, Arnold CD, Cummins J, Khan MSA, Maalouf-Manasseh Z, Siddiqui Z, Ullah MB, Vosti SA Lipid-based nutrient supplementation in the first 1000 d improves child growth in Bangladesh: a cluster-randomized effectiveness trial. Am J Clin Nutr 2017;105:944–57. 28275125 10.3945/ajcn.116.147942

[23] Maxwell D, Ahiadeke C, Levin C, Armar-Klemesu M, Zakariah S, Lamptey GM Alternative food-security indicators: revisiting the frequency and severity of “Coping strategies”. Food Policy 1999;24:411–29.

[24] Šidák Z Rectangular confidence regions for the means of multivariate normal distributions. J Am Stat Assoc 1967;62:626–33.

[25] Streiner DL Best (but oft-forgotten) practices: the multiple problems of multiplicity—whether and how to correct for many statistical tests. Am J Clin Nutr 2015;102:721–8. 26245806 10.3945/ajcn.115.113548

[26] Arimond M, Abbeddou S, Kumwenda C, Okronipa H, Hemsworth J, Jimenez EY, Ocansey E, Lartey A, Ashorn U, Adu-Afarwuah S, Impact of small quantity lipid-based nutrient supplements on infant and young child feeding practices at 18 months of age: results from four randomized controlled trials in Africa. Matern Child Nutr 2017;13:e12377. 10.1111/mcn.12377 PMC5516197 27910260

[27] Hadley C, Tegegn A, Tessema F, Cowan JA, Asefa M, Galea S Food insecurity, stressful life events and symptoms of anxiety and depression in East Africa: evidence from the Gilgel Gibe Growth and Development Study. J Epidemiol Community Health 2008;62:980–6. 18854502 10.1136/jech.2007.068460

[28] Hadley C, Patil CL Food insecurity in rural Tanzania is associated with maternal anxiety and depression. Am J Hum Biol 2006;18:359–68. 16634017 10.1002/ajhb.20505

[29] Natamba BK, Mehta S, Achan J, Stoltzfus RJ, Griffiths JK, Young SL The association between food insecurity and depressive symptoms severity among pregnant women differs by social support category: a cross-sectional study. Matern Child Nutr 2017 Aug 9 [Epub ahead of print; DOI: 10.1111/mcn.12351]. 10.1111/mcn.12351 PMC6865987 27507230

[30] Ali D, Saha KK, Nguyen PH, Diressie MT, Ruel MT, Menon P, Rawat R Household food insecurity is associated with higher child undernutrition in Bangladesh, Ethiopia, and Vietnam, but the effect is not mediated by child dietary diversity. J Nutr 2013;143:2015–21. 24089419 10.3945/jn.113.175182

[31] Choudhury N, Raihan MJ, Sultana S, Mahmud Z, Farzana FD, Haque MA, Rahman AS, Waid JL, Chowdhury AMR, Black RE, Determinants of age-specific undernutrition in children aged less than 2 years—The Bangladesh context. Matern Child Nutr 2017 Oct 12 [Epub ahead of print; DOI: 10.1111/mcn.12362]. 10.1111/mcn.12362 PMC6865922 27731545

[32] Na M, Mehra S, Christian P, Ali H, Shaikh S, Shamim AA, Labrique AB, Klemm RD, Wu LS, West KP Maternal dietary diversity decreases with household food insecurity in rural Bangladesh: a longitudinal analysis. J Nutr 2016;146:2109–16. 27581578 10.3945/jn.116.234229

[33] Frongillo EA, Nguyen HT, Smith MD, Coleman-Jensen A Food insecurity is associated with subjective well-being among individuals from 138 countries in the 2014 Gallup World Poll. J Nutr 2017;147:680–7. 28250191 10.3945/jn.116.243642

